# Supplementary material for: Dynamic infrared aurora on Jupiter
Source: Nat Commun. 2025 May 12;16:3907. doi: 10.1038/s41467-025-58984-z (PMC12069571; doi:10.1038/s41467-025-58984-z)
Supplement: Supplementary file 1 — Supplementary Info [file 41467_2025_58984_MOESM1_ESM.pdf]

Dynamic infrared aurora on Jupiter
Supplementary Information

J. D. Nichols[1*], O. R. T. King[1], J. T. Clarke[2], I. de Pater[3], L. N. Fletcher[1], H. Melin[1,4], L. Moore[2], C. Tao[5], T. K. Yeoman[1]

[1*]School of Physics and Astronomy, University of Leicester, University Road, Leicester, LE1 7RH, Leicestershire, UK.
[2]Center for Space Physics, Boston University, 725 Commonwealth Avenue, Boston, 02215, MA, USA.
[3]Department of Astronomy, University of California, Berkeley, 501 Campbell Hall, Berkeley, 94720, CA, USA.
[4]Department of Mathematics, Physics and Electrical Engineering, Northumbria University, Newcastle-upon-Tyne, Tyne and Wear, UK.
[5]Space Environment Laboratory, National Institute of Information and Communications Technology, Koganei, Japan.

*Corresponding author(s). E-mail(s): jdn4@le.ac.uk;

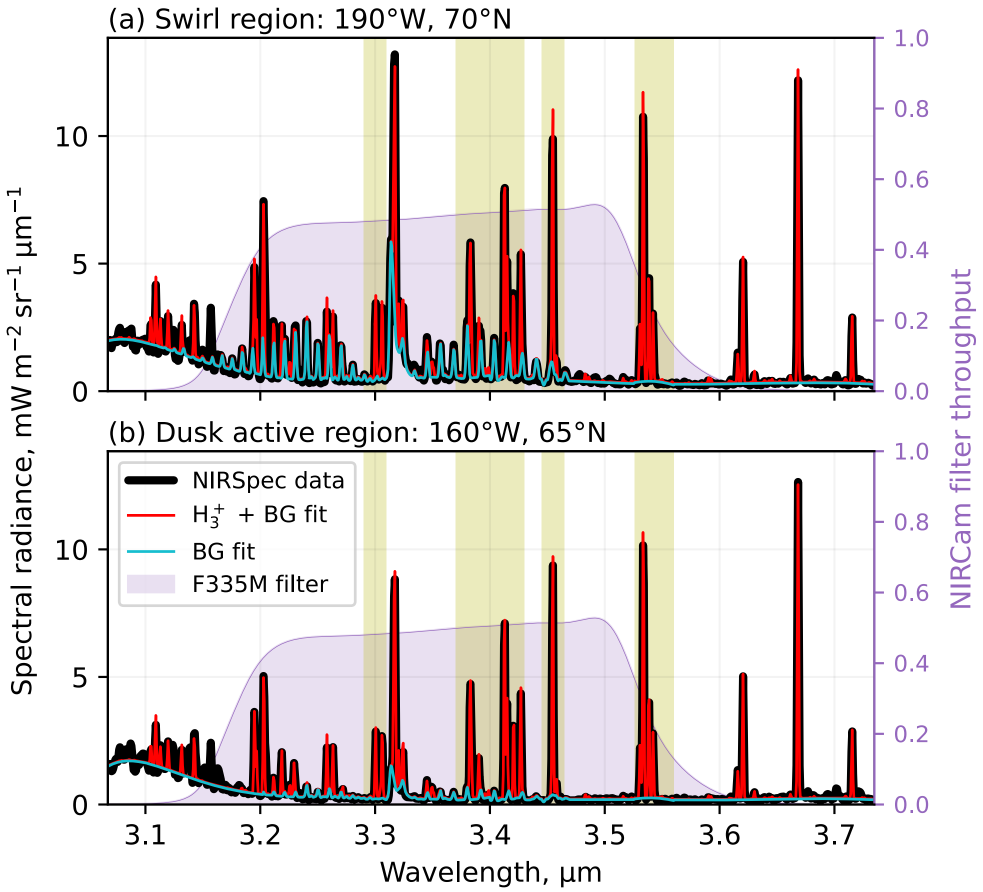

Supplementary Figure 1 Plots showing NIRSpec spectra in the wavelength region of the NIRCam F335M filter from two auroral regions. Panel (a) shows the spectral radiance from 190° W and 70° N, i.e in the Swirl region and panel (b) shows the spectral radiance from 160° W and 65° N, i.e. from the DAR region. In both panels, the observed spectra are shown in black, the overall h3ppy H_3^+ plus background (BG) fit is shown in red, the background alone is shown in cyan, and the NIRCam F335M filter throughput is shown in purple (right hand axis). The regions highlighted in yellow show the wavelength ranges over which the spectral lines were fitted with h3ppy. Data are from observations X. Source data are available as a Source Data file.

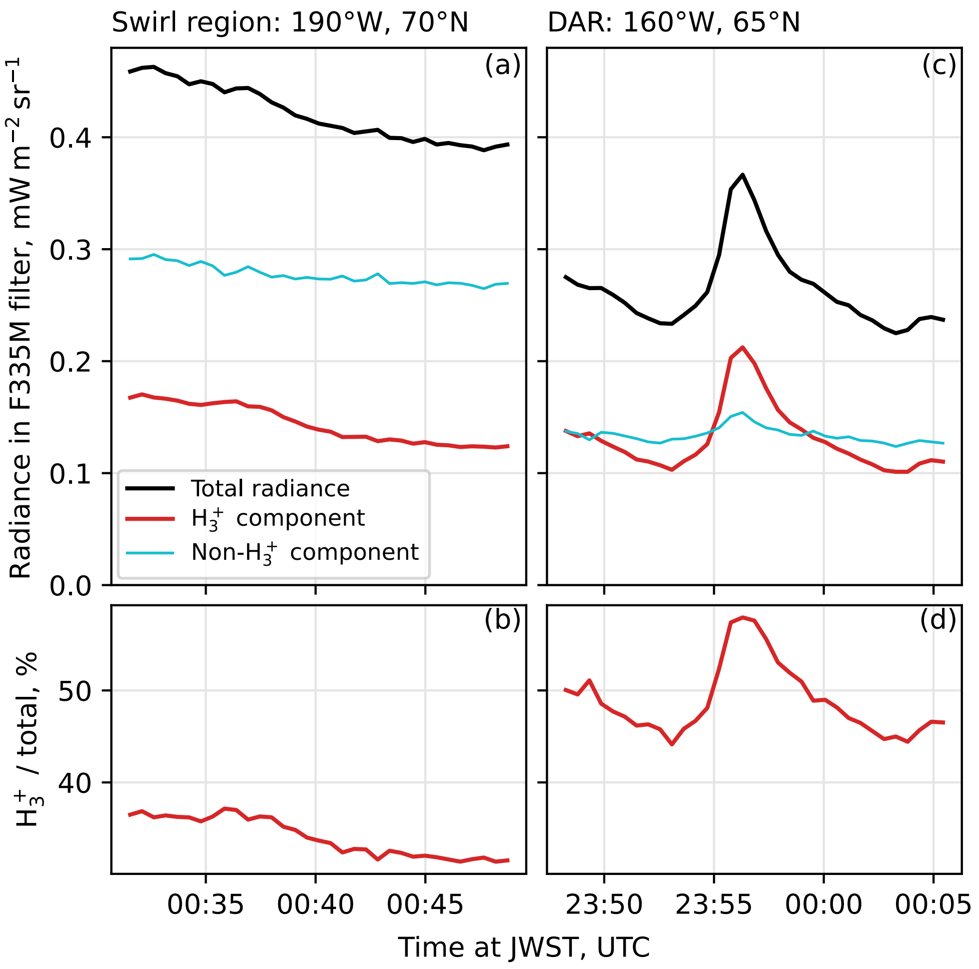

Supplementary Figure 2 Plots of light curves showing the variation in contribution to the total radiance from H_3^+ and non-H_3^+ components for the same regions as Figure 1. Panel (a) shows the total radiance (black), the H_3^+ (red) and non-H_3^+ (cyan) contributions from 190° W and 70° N, i.e in the Swirl region versus time. Panel (b) shows the H_3^+ fraction as a percentage. Panels (c) and (d) show the same for 160° W and 65° N, i.e. from the DAR region. Data are from observations X. Source data are available as a Source Data file.

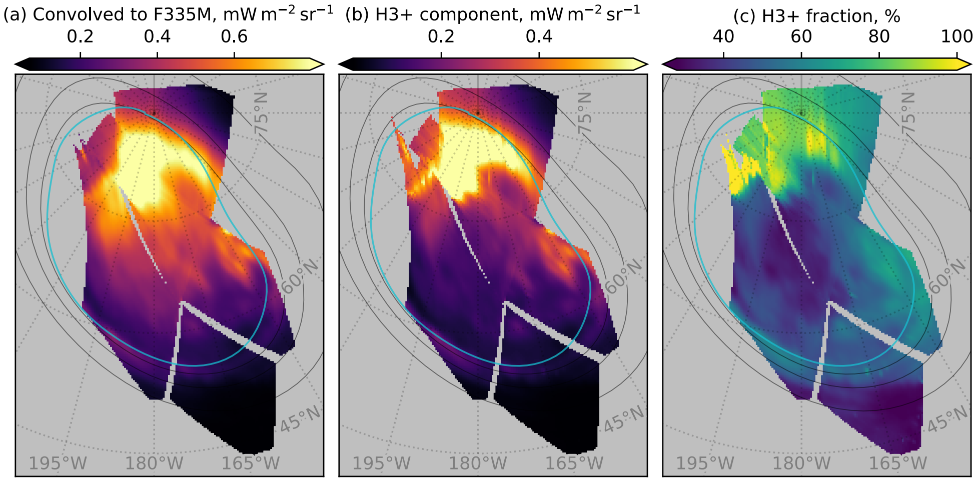

Supplementary Figure 3 Plots showing the spatial variation of the H_3^+ contribution to the radiance in the F335M filter window. Panel (a) shows the mean NIRSpec spectral radiance at each location convolved with the F335M filter throughput. Panel (b) shows the H_3^+ radiance, and panel (c) shows the fraction of the total radiance that is from H_3^+. Images are presented in the same format as Fig. 7. Data are from all NIRSpec observations. Source data are available as a Source Data file.

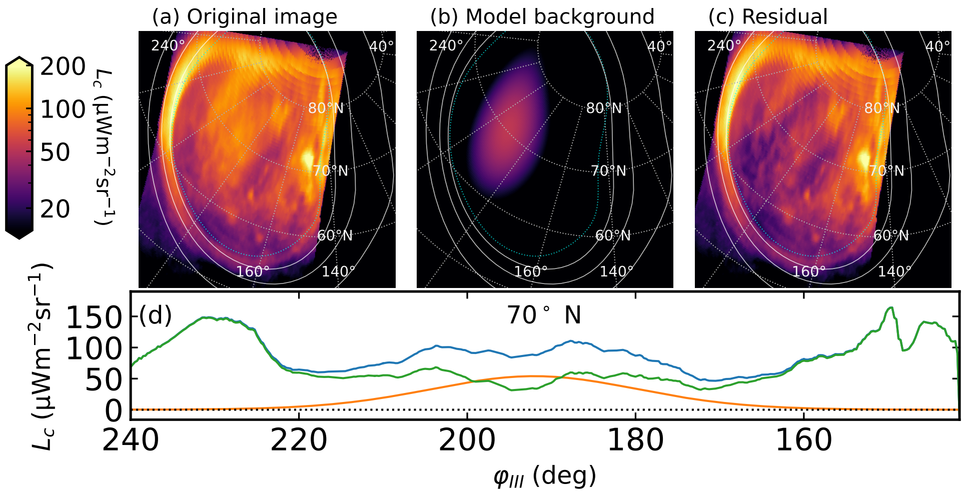

Supplementary Figure 4 Plots showing the model methane background and its subtraction. Panel (a) shows a representative original image radiance L_c (from observation 3104) projected in the same format as Fig. 2. Panel (b) similarly shows the model methane non-LTE emission background. Panel (c) shows the residual radiance following subtraction of the background, and panel (d) shows radiance profiles along 70° N latitude versus System III longitude φ_{III}. In panel (d), the blue line is the original, orange is the model and green is the residual. Source data are available as a Source Data file.

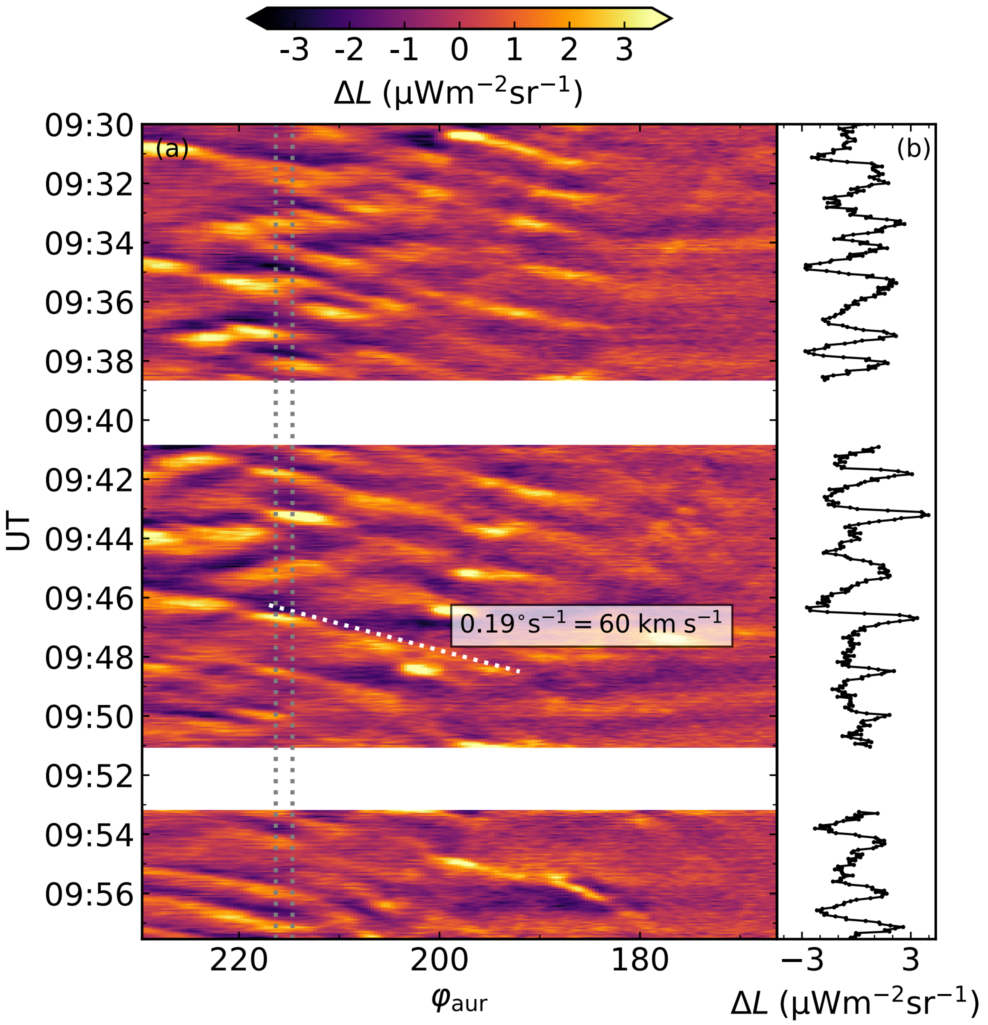

Supplementary Figure 5 Plot illustrating the rapid eastward-travelling auroral pulses (REAPs) in the dark polar region (DPR). Panel (a) shows a keogram, i.e. residual radiance ΔL as a function of auroral longitude φ_{aur} and time UT. Panel (b) shows a cut taken from between the vertical grey dotted lines in panel (a). The white dotted line in panel (a) illustrates the slope shown in the white box. Source data are available as a Source Data file.

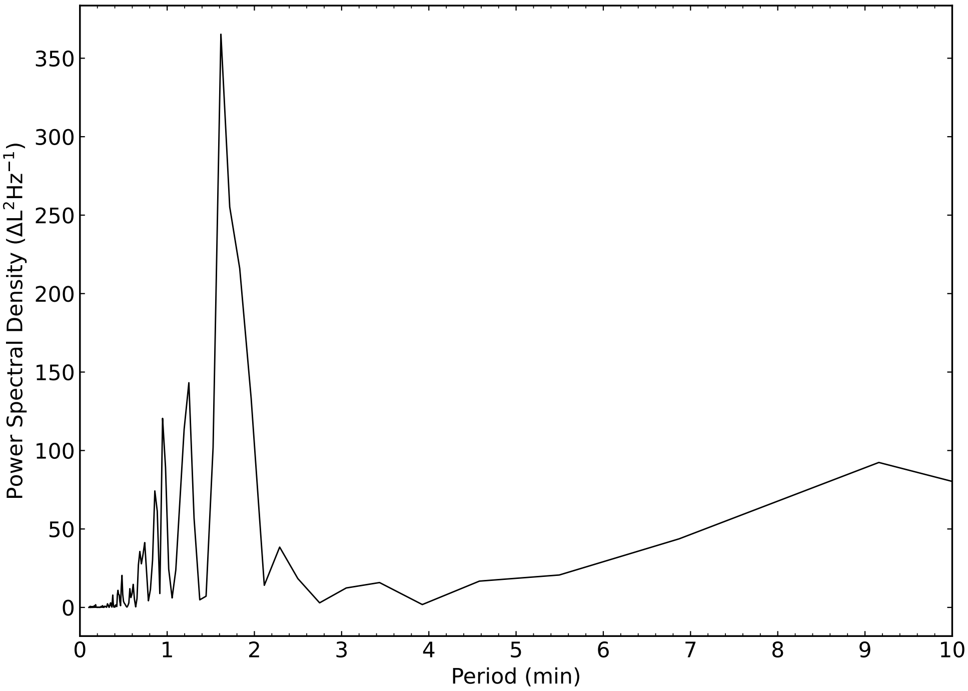

Supplementary Figure 6 Periodogram of the keogram slice shown in Supplementary Figure 5b. Specifically, the power spectral density of the residual radiance ΔL versus period in minutes. Source data are available as a Source Data file.

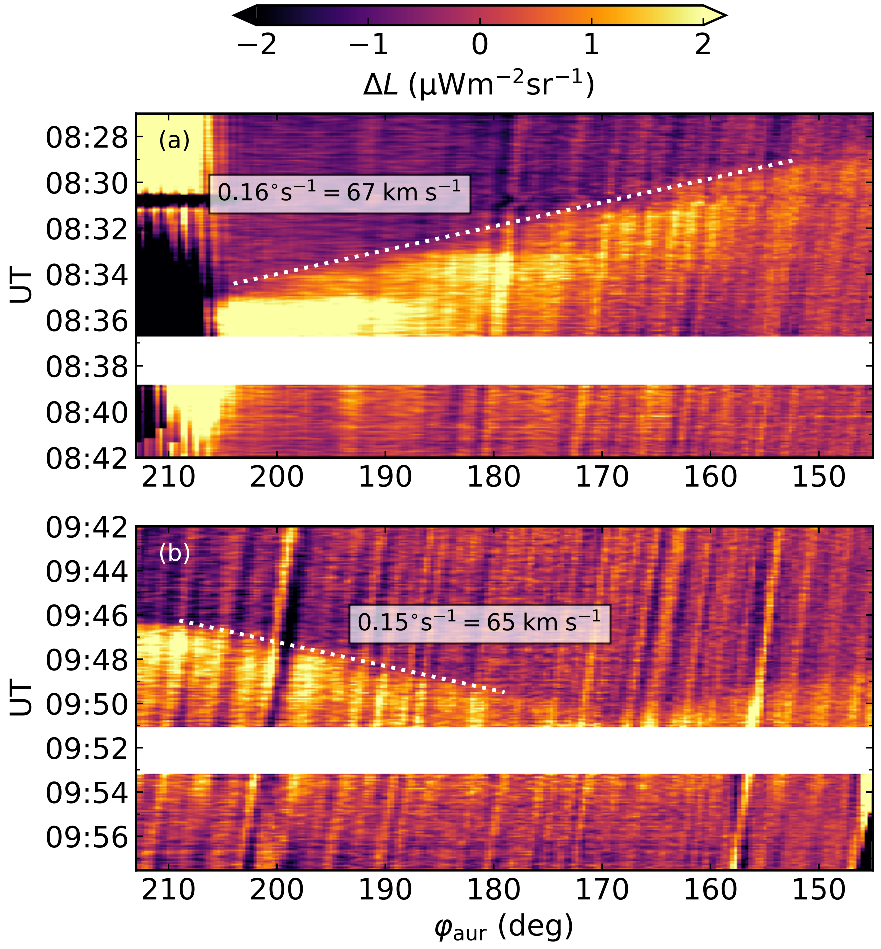

Supplementary Figure 7 Plot illustrating rapid longitudinal pulsations propagating along the Io footprint (IFP) tail. Keograms of residual radiance ΔL in the IFP tail versus auroral longitude φ_{aur} and time UT. Panel (a) shows a westward-propagating pulsation, and panel (b) shows two, with an eastward pulsation highlighted. The white dotted lines illustrate the slopes shown in the white boxes. Source data are available as a Source Data file.
