# Supplementary material for: Dynamic infrared aurora on Jupiter
Source: Nat Commun. 2025 May 12;16:3907. doi: 10.1038/s41467-025-58984-z (PMC12069571; doi:10.1038/s41467-025-58984-z)
Supplement: Supplementary file 2 — Description of Additional Supplementary Files [file 41467_2025_58984_MOESM2_ESM.docx]

**Description of Additional Supplementary Files**

File Name: Supplementary Movie 1
Description: **All NIRCam images of Jupiter's northern auroral H_3_^+^ emission radiance *L* obtained in Program 4566**. The left panel shows the 3s integrations, as observed, such that dawn is to the left, dusk to the right, and noon toward the bottom. The start time in universal time (UT) is shown at the top, both at Earth (E) and corrected for one-way light travel time (J). The planet's limb (grey solid line) and a 10° × 10° graticule (grey dotted lines) at the 1-bar altitude are overlaid, along with solid lines which show the mapped moon footprints (respectively, with increasing latitude Io, Europa and Ganymede) [Connerney et al. 2022] and reference main oval [Nichols et al., 2017] (cyan dotted line) plotted at 500 km above the 1-bar level. The right panel shows the line-of-sight corrected radiance *L_c_* values projected onto a spheroid 500 km above the 1-bar level and plotted in a polar stereographic projection. A 20° × 10° graticule is overlaid in grey, along with solid lines which show the mapped moon footprints and reference main oval (cyan dotted line). The coordinate system is planetocentric, labelled with System III westward longitude. The central meridian longitude is oriented toward the bottom. Source data are available on Figshare [Nichols, 2024].

File Name: Supplementary Movie 2
Description: **Simultaneous far-ultraviolet (FUV) and near-infrared (NIR) auroral emission energy flux on Jupiter.** The left panel shows the H_2_ energy flux $E_{H_{2}}$ from HST observation of8v02dmq, while right panel shows the H_3_^+^ energy flux $E_{H_{3}^{+}}$ from JWST observations NIRCam3105, NIRCam3106, NIRCam3107 and NIRCam3108. In each case, both images were obtained with the same start time. Images are shown projected onto a spheroid 240 km (500 km) above the 1-bar level for the FUV (NIR) emission and plotted in a polar stereographic projection. A 20° × 10° graticule is overlaid in grey, along with solid lines which show the mapped moon footprints and reference main oval (red and cyan dotted lines for FUV and NIR, respectively). The coordinate system is planetocentric, labelled with System III westward longitude. The central meridian longitude is oriented toward the bottom. The identical red and cyan solid- (dashed-) line boxes show the regions from which the energy fluxes shown in Fig. 5 (Fig. 6) are derived. Specifically, these are the dusk active region (DAR), noon active region (NAR), and two regions of the main emission (ME1 and ME2) as labelled. Source data are available on Figshare [Nichols, 2024].
